# Proteomics Analysis on the Effects of Oxidative Stress and Antioxidants on Proteins Involved in Sterol Transport and Metabolism in Human Telomerase Transcriptase-Overexpressing-Retinal Pigment Epithelium Cells

**DOI:** 10.3390/ijms252010893

**Published:** 2024-10-10

**Authors:** R. Scott Duncan, Andrew Keightley, Adam A. Lopez, Conner W. Hall, Peter Koulen

**Affiliations:** 1 Vision Research Center, Department of Ophthalmology, School of Medicine, University of Missouri—Kansas City, 2411 Holmes St., Kansas City, MO 64108, USAconnerhall1212@gmail.com (C.W.H.); 2 Department of Biomedical Sciences, School of Medicine, University of Missouri—Kansas City, 2411 Holmes St., Kansas City, MO 64108, USA

**Keywords:** age-relate macular degeneration (AMD), retinal pigment epithelium (RPE), oxidative stress, vitamin E, tocopherol, antioxidant, proteomics, sterol

## Abstract

Age-related macular degeneration (AMD) is the most prevalent ocular disease in the elderly, resulting in blindness. Oxidative stress plays a role in retinal pigment epithelium (RPE) pathology observed in AMD. Tocopherols are potent antioxidants that prevent cellular oxidative damage and have been shown to upregulate the expression of cellular antioxidant proteins. Here, we determined whether oxidative stress and tocopherols, using either normal cellular conditions or conditions of sublethal cellular oxidative stress, alter the expression of proteins mediating sterol uptake, transport, and metabolism. Human telomerase transcriptase-overexpressing RPE cells (hTERT-RPE) were used to identify differential expression of proteins resulting from treatments. We utilized a proteomics strategy to identify protein expression changes in treated cells. After the identification and organization of data, we divided the identified proteins into groups related to biological function: cellular sterol uptake, sterol transport and sterol metabolism. Exposure of cells to conditions of oxidative stress and exposure to tocopherols led to similar protein expression changes within these three groups, suggesting that α-tocopherol (αT) and γ-tocopherol (γT) can regulate the expression of sterol uptake, transport and metabolic proteins in RPE cells. These data suggest that proteins involved in sterol transport and metabolism may be important for RPE adaptation to oxidative stress, and these proteins represent potential therapeutic targets.

## 1. Introduction

Steroid hormones play a key role in many physiological functions, including reproduction, digestion, water homeostasis and response to stress, among many others [1,2,3,4,5]. Cholesterol, a sterol, is a molecular substrate for steroid hormone and bile acid synthesis [6]. De novo synthesis of cholesterol takes place in the ER due to the enzymes in the mevalonate pathway and diet [6]. In circulation, low-density lipoprotein–low-density lipoprotein receptor (LDL-LDLR) plasma membrane complexes are endocytosed and are processed to release free cholesterol. Within the cell, cholesterol is converted by a cholesterol side chain cleavage enzyme, cytochrome P450 family 11A1, in mitochondria into pregnenolone, the precursor for all steroid hormones [7]. Another cytochrome P450 enzyme, CYP17A1 (17-alpha-hydroxylase), converts pregnenolone to 17-alpha-hydroxypregnenolone in the ER [8]. Hydroxysteroid 17-beta dehydrogenase 3 (HSD17B3) converts pregnenolone to progesterone and 17-alpha-hydroxypregnenolone to 17-alpha-hydroxyprogesterone in the ER. The previously mentioned enzymes also produce androgens, and aromatase (CYP19A1) converts androgens to estrogens [6,8].

Steroid hormones play a critical role in central nervous system (CNS) functions such as neuronal differentiation and neuroprotection [9,10]. The retina, a CNS tissue, also requires steroid hormones to properly function [3]. Estrogens and androgens are known to be produced in retinal tissue [11], and estrogen, or a lack thereof, may play a role in multiple ocular diseases such as cataracts, glaucoma and age-related macular degeneration (AMD) [12]. In the retina, steroids can exhibit neuroprotective properties and can regulate ocular blood flow. Progestin exposure has been shown to slow the progression of retinitis pigmentosa [11].

The concentration of various steroid hormones in the blood differs between males and females. Estrogen (E2) concentrations can affect visual function in females of advanced age. For example, macular damage is more common among postmenopausal women, likely due to the rapid postmenopausal decrease in estrogen synthesis [13]. Estrogen exposure may be protective against AMD, but this depends on several factors such as age at menopause, pregnancy number, oral contraceptive use and hormone replacement therapy (HRT) [11,12,13]. A recent prospective case–control study revealed that, after adjustment for age and race, subjects with AMD exhibited a greater likelihood of having received less HRT than those with higher HRT use [13]. Other studies suggest that in females, a greater lifetime estrogen exposure may be linked to a greater likelihood of having wet AMD [14].

Oxidative stress is a contributing factor to the progression and severity of AMD [15]. The Age-Related Eye Diseases (AREDs) human clinical trials were carried out in part to determine if administration of antioxidant compounds, including vitamin E, reduced the severity of AMD and slowed AMD progression. Antioxidant (AO) administration only delayed the advancement of severe macular degeneration [16]. To further explore the utility of antioxidant protection of RPE using an in vitro direct application approach, we determined what effect the oxidant, tertbutyl hydroperoxide (tBHP) and/or α-tocopherol (αT) or γ-tocopherol (γT) on the levels of proteins regulating steroid hormone transport and metabolism in human telomere reverse transcriptase overexpressing RPE (hTERT-RPE) cells.

In this study, we treated hTERT-RPE cells with αT or γT (or DMSO vehicle) for 24 h followed by a replacement with media containing either the oxidant, tertbutyl hydroperoxide (tBHP) or vehicle for another 24 h. We conducted a proteomics study on these six treatment groups and identified a differential expression of proteins mediating steroid hormone (sterol) transport and metabolism.

## 2. Results

**Figure 1:**
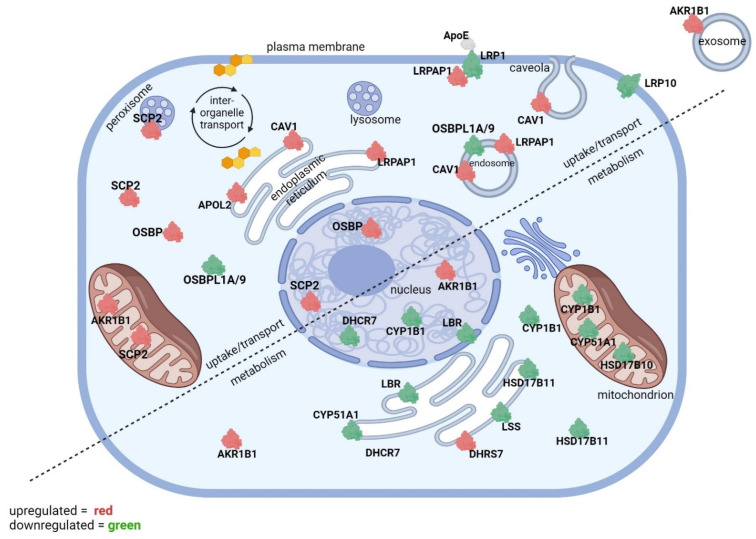
**The effect of tBHP-mediated oxidative stress on the expression of proteins involved in sterol uptake/transport and metabolism.** Upregulated (red) and downregulated proteins from Tables 1, 3 and 5 are displayed in a diagram of an RPE cell. The diagram is divided into two segments—proteins involved in sterol uptake and transport (top and left) and proteins involved in sterol metabolism (bottom and right). Subcellular localizations for differentially expressed proteins are included.

**Figure 2:**
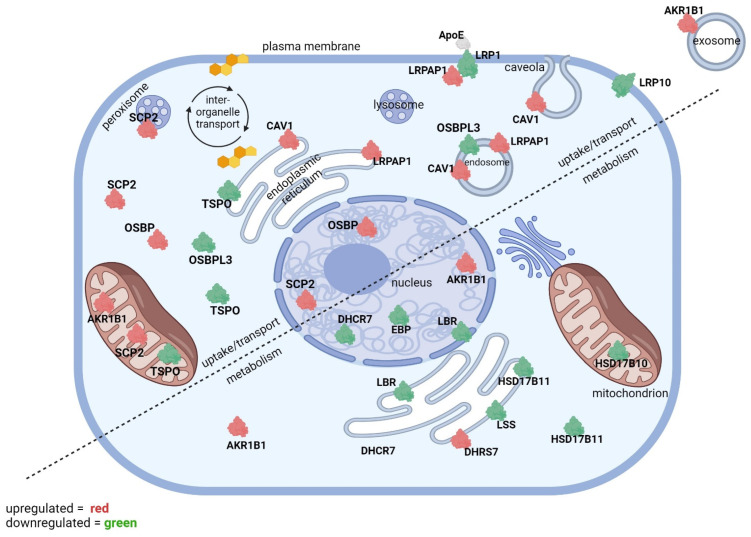
**The effect of αT and γT exposure on the expression of proteins involved in sterol uptake/transport and metabolism.** Upregulated (red) and downregulated proteins from Tables 2, 4 and 6 are displayed in a diagram of an RPE cell. The diagram is divided into two segments—proteins involved in sterol uptake and transport (top and left) and proteins involved in sterol metabolism (bottom and right). Subcellular localizations for differentially expressed proteins are included.

There was a total of 4174 proteins identified in this study, and 1828 proteins (44%) were differentially expressed between the control and tBHP-exposed groups with *p* ≤ 0.050. The number of proteins differentially expressed in the tocopherol groups compared to vehicle, including the tocopherol-tBHP groups compared to veh-tBHP, were lower (<25% of the total at *p* ≤ 0.050). From our proteomic dataset (first published in Duncan et al., 2023 [17], we generated a list of GO biological function categories containing a list of differentially expressed proteins related to sterol uptake, transport and metabolism by searching for the terms ‘sterol’, ‘oxysterol’, ‘steroid’, ‘steroid hormone’, ‘low-density lipoprotein’, ‘high-density lipoprotein’ and ‘apolipoprotein’. The top GO biological function categories (combined score ≥ 2.0) are relevant to sterol uptake, transport and metabolism. Cholesterol binds to various proteins involved in cholesterol uptake and transport proteins for import into the cell as well as intracellular transport for steroidogenesis in mitochondria. Exposure of hTERT-RPE cells to tocopherol or tBHP for 24 h led to changes in 22 proteins implicated in sterol transport and sterol metabolism (Figure 1). These proteins are divided into three broad categories—cellular sterol uptake, intracellular sterol transport and sterol metabolism. The cellular exposure to αT or γT led to the up- and downregulation of many of the same proteins affected by tBHP exposure (Figure 2).

### 2.1. Cellular Sterol Uptake

The cellular uptake of cholesterol occurs via endocytosis of lipoproteins such as low-density lipoproteins (LDL) and very low-density lipoproteins (VLDL). LDL/VLDL uptake requires LDL/VLDL receptors such as LDL receptor, LDL receptor-related proteins (LRPs), CD36 and others. Exposure to veh/tBHP increased the expression of LDL receptor-related protein-associated protein 1 (LRPAP1; +20.37-fold, *p* ≤ 0.01) and apolipoprotein L2 (APOL2; +17.0-fold, *p* = 0.022) (Table 1). LRPAP1 is a protein that mediates low-density lipoprotein (LRP) chaperone, mediating proper folding and targeting the proper subcellular location [18]. LRPAP1 interacts with LRP1, LRP2 and LDLR to mediate internalization, and it prevents LRP1 interaction with alpha-2-macroglobulin [19]. APOL2 is localized in the cytosol, where it mediates the transport of cholesterol (and other lipids) to the PM and various organelles [20]. LRP10 is a receptor that plays a role in the uptake of extracellular lipophilic molecules, including APOE and intracellular signaling [21].

Exposure to veh/tBHP downregulated low-density lipoprotein receptor-related protein 10 (LRP10; −6.4-fold, *p* ≤ 0.01) and low-density lipoprotein receptor-related protein 1 (LRP1; −15.1-fold, *p* ≤ 0.01) (Table 1). LRP10 is a receptor mediating the cellular uptake of lipophilic molecules and possibly APOE in the liver. LRP1 plays a role in numerous processes within the cell, including the maintenance of lipid homeostasis and signal transduction [21,22]. LRP1 is an APOE and alpha-2-macroglobulin receptor that plays a role in clearing plasma cholesterol [19]. The differential regulation of proteins involved in sterol uptake mediated by oxidative stress indicates that less cholesterol uptake may be a cellular response to mitigate the effects of oxidative stress.

hTERT-RPE cell exposure to αT/NT or γT/NT upregulated the expression of LRPAP1 (+19.9-fold, *p* ≤ 0.01 and +17.5-fold, *p* ≤ 0.01, respectively) (Table 2). Neither αT/NT nor γT/NT affected APOL2 expression, suggesting that oxidative stress may be a specific requirement for APOL2 induction. The exposure of cells to αT/NT had no effect on LRP10 or LRP1, but exposure to γT downregulated LRP10 (−4.8-fold, *p* ≤ 0.01) and LRP1 (−9.2-fold, *p* ≤ 0.01) (Table 2). The differential regulation of proteins involved in sterol uptake in response to tocopherol (antioxidant) exposure suggests that less cholesterol uptake may be a cellular response to better prepare for the effects of oxidative stress.

### 2.2. Intracellular Sterol Transport

Sterols and steroid hormones are transported intracellularly via cholesterol/sterol transport proteins [23,24,25]. Veh/tBHP exposure upregulated the expression of sterol carrier protein 2 (SCP2; +42.8-fold, *p* ≤ 0.01), caveolin 1 (CAV1; +20.6-fold, *p* ≤ 0.01) and oxysterol binding protein (OSBP; +6.6-fold, *p* = 0.020) (Table 3). SCP2 is a non-specific lipid transport protein that transports cholesterol between the ER and plasma membrane (PM) [25,26]. Cholesterol is transported to the inner mitochondrial membrane, where it is oxidized (hydroxylated) to the steroid pregnenolone [25]. SCP2 has been shown to increase the synthesis of cholesterol to 7-DHC to initiate the synthesis of steroids [26]. Caveolin 1 (CAV1) is a molecular scaffold that is a major constituent of the PM that couples integrins to MAPK pathway signaling [27]. CAV1 can directly bind cholesterol and regulate proteins involved in cholesterol efflux (ABCG1) and with steroid hormone receptors and coactivators [28]. CAV1 expression is regulated by intracellular cholesterol levels [29]. OSBP can transport sterols from lysosomes to the nucleus [24]. OSBP plays a role in the transport of lipids between the Golgi and ER membranes [24].

Exposure to veh/tBHP led to a decrease in the expression of oxysterol binding protein like 1A (OSBPL1A; −5.1-fold, *p* = 0.011) and oxysterol binding protein like 9 (OSBPL9; −11.6, *p* = 0.014) (Table 3). Oxysterol binding protein like 1A (OSBPL1A) may be an intracellular lipid transporter involved in bile biosynthesis [30]. OSBPL1A transports 25-hydroxycholesterol (25-OH-cholesterol) and cholesterol from lysosomal compartments to the nucleus and between the Golgi and the ER [24,31]. OSBPL9 is also a cholesterol transporter that aids in maintaining Golgi structure, and it may regulate bile salt synthesis [32]. The differential regulation of proteins involved in sterol transport in response to oxidative stress suggests that sterol trafficking may be a cellular response to mitigate the effects of oxidative stress.

Like veh/tBHP, exposure of cells to αT/NT or γT/NT upregulated SCP2 (+31.5-fold, *p* = 0.012 and +50.5-fold, *p* ≤ 0.01, respectively) and OSBP (+5.4-fold, *p* = 0.049 and +13.0-fold, *p* ≤ 0.01, respectively) (Table 4). Veh/tBHP exposure had no effect on OSBPL3 expression, but exposure to αT or γT downregulated the expression of OSBPL3 (−15.9-fold, *p* = 0.032 and −14.9-fold, *p* = 0.037, respectively). Similarly, veh tBHP exposure had no effect on translocator protein (TSPO), but exposure to αT or γT downregulated TSPO (−41.0-fold, *p* ≤ 0.01 and −43.0-fold, *p* ≤ 0.01, respectively) (Table 4).

Like OSBPL1A, OSBPL3 binds 25-OH-cholesterol, and cholesterol transports sterols between the PM and ER, which is involved in bile synthesis [31,33]. TSPO is predominantly expressed in the ‘transducosome’ complex in the mitochondrial outer membrane and is important in the mitochondrial import of cholesterol [34]. Cholesterol accumulation in macrophages elevated TSPO expression and upregulated the expression of genes involved in cholesterol efflux [34].

Studies in RPE cells revealed that TSPO deficiency led to reduced efflux of cholesterol and elevated levels of reactive oxygen species [35]. Intracellular cholesterol accumulation was elevated in TSPO knockout RPE cells. Aging RPE exhibits a decrease in TSPO expression with diminished cholesterol efflux [35].

### 2.3. Sterol Transport Proteins Present but Not Significantly Changed

We identified several proteins that are involved in sterol transport, metabolism and signaling, but they were not differentially expressed following our treatments. We included these proteins as their reported presence aids in fully understanding what sterol/steroid-related proteins may be important for RPE function. OSBPL11, another oxysterol binding protein, may bind to 25-OH-cholesterol [31]. OSBPL8 binds cholesterol and oxysterols and mediates their transfer between the ER and PM [36,37]. Niemann-Pick C1 Protein (NPC1) is localized to the endosome/lysosome compartments to facilitate cholesterol transport. NPC1 also traffics LDLs to endosomes or lysosomes, where they become degraded and where cholesterol is released. [38,39].

### 2.4. Cholesterol/Steroid Metabolism

Cells exposure to tBHP (veh-tBHP) increased the expression of dehydrogenase/reductase 7 (DHRS7; +22.0-fold, *p* ≤ 0.01) and aldo-keto reductase 1B (AKR1B1; +19.5-fold, *p* ≤ 0.01) (Table 5). DHRS7 is an oxidoreductase that reduces carbonyl moieties on multiple substrates, such as steroids [40,41]. For example, DHRS7 reduces 5α-dihydrotestosterone to 3α-androstanediol, thereby regulating androgen receptor activity [42]. In vitro, DHRS7 reduces cortisone to 20β-dihydrocortisone [42]. AKR1B1 reduces multiple compounds containing carbonyl groups, including sterols [43].

Exposure of cells to tBHP (veh-tBHP) reduced the expression of hydroxysteroid-17β-dehydrogenase 10 (HSD17β10; −8.6-fold, *p* ≤ 0.01), lanosterol synthase (LSS; −9.5, 0.013), hydroxysteroid-17β-dehydrogenase 11 (HSD17β11; −13.6-fold, *p* = 0.048), lamin B receptor (LBR; −17.2, *p* = 0.017), 7-dehydrocholesterol reductase (DHCR7; −20.5-fold, *p* ≤ 0.01), cytochrome P450 family 1B1 (CYP1B1; −21.5-fold, *p* ≤ 0.01) and cytochrome P450 family 51A1 (CYP51A1; −27.7-fold, *p* = 0.011) (Table 5).

HSD17β10 is a mitochondrial dehydrogenase that mediates fatty acid and steroid metabolism [44,45]. HSD17β10 can oxidize multiple hydroxysteroids as well as some hydroxylated bile acids [44,45]. LSS cyclizes oxidosqualene to lanosterol, which is the initial step in synthesizing cholesterol, steroid and vitamin D.

Hydroxysteroid dehydrogenase 17β member 11 (HSD17B11) mediates the oxidation of steroid hormones [46]. HSD17B11 converts androstanediol to androsterone [47]. LBR couples the nuclear lamina and chromatin with the inner nuclear membrane, but it also reduces lanosterol to mediate the synthesis of cholesterol [48]. LBR plays an important role in the synthesis of cholesterol, and it can regulate cholesterol levels needed for the formation of lipid rafts in membranes [49,50].

DHCR7 carries out the last step of cholesterol synthesis—the conversion of 7-dehydrocholesterol (7-DHC) to cholesterol [51]. DHCR7 is expressed in the ER and outer nuclear membranes. DHCR7 gene mutations can lead to decreased levels of serum cholesterol and increased levels of serum 7-DHC [51,52]. DHCR7 has been shown to interact with CYP51A1 [52]. Human phenotypes associated with DHCR7 include vitamin D concentration [51].

Cytochrome P450 1 B1 (CYP1B1) is a monooxygenase that plays a major role in the metabolism of fatty acids and steroid hormones [53]. CYP1B1 is located in the ER where it oxidizes 17beta-estradiol to generate hydroxyestrogens from estrone and 17beta-estradiol [53]. CYP1B1 also hydroxylates testosterone and progesterone [54]. Cytochrome P450 51 A1 (CYP51A1) is an enzyme that plays a role in the metabolism of steroids and cholesterol synthesis in the ER by demethylating lanosterol and 24,25-dihydrolanosterol [55].

Similar to that of tBHP exposure, exposure of cells to αT or γT altered the expression of AKR1B1 (+25.6-fold, *p* ≤ 0.01 for αT and +18.9-fold, *p* ≤ 0.01 for γT), HSD17B10 (−16.0-fold, *p* = 0.033 for αT and −7.7-fold, *p* = 0.016 for γT)) and DHCR7 (−22.3-fold, *p* = 0.011 for αT and −25.7, *p* = 0.003 for γT) (Table 6). Unlike that for tBHP exposure, exposure to αT or γT reduced the expression of DHRS4 (−12.2-fold, *p* = 0.044 for αT and −16.0-fold, *p* = 0.017 for γT) (Table 6).

Unlike that for tBHP exposure, exposure to αT or γT has no effect on DHRS7, CYP51A1 and CYP1B1 expression. Cell exposure to γT, but not αT, downregulated lanosterol synthetase (LSS; −16.3-fold, *p* ≤ 0.01) and HSD17B11 (−14.0-fold, *p* = 0.045) to a greater degree than that of tBHP (Table 6). Exposure of cells to γT, but not tBHP or αT, downregulated ergosterol binding protein (EBP; −15.8-fold, *p* = 0.026 for γT versus −12.7-fold, 0.052 for tBHP and −9.5-fold, *p* = 0.116 for αT) (Table 6).

### 2.5. The Effect of αT or γT Pretreatment on tBHP-Mediated Changes in Protein Expression

In addition to uncovering the effect of tBHP, αT and γT, we identified which proteins from these treatment groups were up- or downregulated by the dual αT-tBHP or γT-tBHP treatments compared to veh-tBHP alone. There are three types of effects represented here—(1) αT and/or γT pretreatment has no effect on tBHP-mediated up- or downregulation of protein expression, (2) αT and/or γT lessens/reverses the tBHP-mediated effect, and (3) αT and/or γT pretreatment potentiates the tBHP-mediated effect.

There were twelve proteins differentially expressed in response to tBHP (veh-tBHP) but unaffected by pretreatment with αT or γT—APOL2, LRP10, OSBP, OSBP3, OSBP9, HSD17B10, HSD17B11, LSS, DHRS4, DHCR7, CYP1B1 and CYP51A1 (Table 7). There were nine differentially expressed proteins in the αT-tBHP or γT-tBHP-exposed condition compared to veh-tBHP exposed alone (Table 7). Specifically, there were two proteins from the cellular sterol uptake group, four from the sterol transport group, and three from the sterol metabolism group. The pre-exposure of cells to αT or γT followed by tBHP resulted in an 8.3-fold and 3.7-fold loss of LRPAP1 upregulation mediated by veh-tBHP, respectively (Table 7). Similarly, pre-exposure of cells to αT prior to tBHP exposure led to a 20.8-fold exposure to γT followed by tBHP and 7.8-fold loss of CAV1 upregulation and a 3.9-fold (αT) and 5.6-fold (γT) loss in tBHP mediated SCP2 upregulation mediated by veh-tBHP alone. Pre-exposure of cells to αT, but not γT, followed by tBHP led to a 6.5-fold loss of DHRS7 upregulation mediated by veh-tBHP alone. Pre-exposure to αT or γT (αT-NT or γT-NT) led to a trend toward the potentiation of tBHP-mediated downregulation, but it failed to reach statistical significance (Table 7; −2.8-fold, *p* = 0.055 for αT-tBHP/veh-tBHP and −2.7-fold, *p* = 0.056 for γT).

There were differences between αT and γT effects on tBHP-mediated changes in protein expression. For example, αT-NT exposure had no effect on DHRS7 expression, but αT pre-exposure lessened the tBHP-mediated upregulation of DHRS7. Although tBHP had no effect on TSPO expression, αT, but not γT, still upregulated the TSPO expression in the presence of tBHP.

Pre-exposure of cells to γT, but not αT, reversed the tBHP-mediated downregulation of OSBPL1A expression (+5.3-fold, *p* = 0.038) (Table 7). In addition, γT, but not αT, exposure followed by tBHP exposure potentiated the upregulation of EBP (+3.6-fold, *p* ≤ 0.01).

### 2.6. Sterol Metabolizing Proteins Present but Not Differentially Expressed

Two proteins involved in sterol metabolism were detected in the treatment groups but were not significantly differentially expressed. These include ferredoxin 1 (FDX1) and neutral cholesterol ester hydrolase 1 (NCEH1). FDX1 is a small iron-sulfur protein that reduces cytochrome P450 in mitochondria to mediate the metabolism of steroids, bile acids and vitamin D [56]. NCEH1 can hydrolyze cholesterol esters [57]. 

There was a trend toward a downregulation in ergosterol biosynthesis 28 homolog (ERG28) in response to γT exposure (−16.0-fold, *p* = 0.063), but not tBHP (−0.6-fold, *p* = 0.759) or αT (−5.9-fold, *p* = 0.420) exposure. ERG28 plays a significant role in sterol biosynthesis [58], and it may interact with several proteins involved in cholesterol/steroid transport or metabolism, including LSS, LBR, CYP51A1, DHCR7 and several 3 beta-hydroxysteroid dehydrogenases (STRING interaction network).

### 2.7. Differential Expression of Proteins More Indirectly Related to Steroid Hormone Function

tBHP exposure upregulated the expression of fatty acid-binding protein 5 (FABP5; +11.7-fold). FABP5 is an intracellular very long chain fatty acid (VLCFA) carrier and transports fatty acids from the cytoplasm into the nucleus [59,60]. While its direct effect on sterol transport is currently unclear, FABP5 inhibition has been shown to affect cholesterol levels in human RPE cells [61]. FABP5 is a fatty acid-binding protein expressed in epidermal cells. FABP5 polymorphisms are associated with type 2 diabetes [61,62]. A reduction in the expression of FABP5 in the RPE/choroidal complex occurs in a murine model of early-stage AMD [61]. FABP5 protein inhibition in human RPE cells led to cholesterol reduction, the presence of lipid droplets and a reduction in the release of APOB [61]. In patients with type 2 diabetes receiving statin therapy, an independent association was found between serum FABP5 concentration and low HDL cholesterol [63].

Exposure to tBHP downregulated ABC binding cassette subfamily D member 1 (ABCD1; −19.3-fold) and solute carrier family 27 member 1 (SLC27A1; −23.3-fold).

Mutations in the ABCD1 gene cause the peroxisomal disorder, X-linked adrenoleukodystrophy (X-ALD), which results in VLCFA accumulation [64]. ABCD1 impairment in fibroblasts isolated from X-ALD patients and CNS tissues of ABCD1-knockout mice was shown to affect the metabolism of cholesterol [64]. There were elevated cholesterol ester levels containing VLCFA. ABCD-deficient fibroblasts exposed to high cholesterol concentrations exhibited elevated conversion of cholesterol to a cholesterol ester as well as increased formation of lipid droplets [64]. On the other hand, NCEH1 expression and ABCA1-mediated cholesterol efflux were increased, as was the progesterone-induced release of cortisol [64].

SLC27A1 facilitates LCFA import into the cell and catalyzes fatty acyl-CoA formation by utilizing LCFA as substrates, and SLC27A1 may regulate cholesterol metabolism [60].

## 3. Discussion

Here, we determined what effect exposure to tBHP, αT γT or both had on the expression of proteins mediating sterol/steroid hormone uptake, transport and metabolism. Our proteomics approach provided novel data on how hTERT-RPE cells respond to these stimuli with respect to sterol/steroid function.

Cholesterol and steroid hormone signaling does not appear to be highly associated with the onset of AMD, but it may contribute to the disease process. In another study, an analysis was carried out on AMD microarray data derived from the Gene Expression Omnibus (GEO) database that revealed the differential expression of over 1000 genes in AMD [65]. After KEGG analysis, the steroid biosynthesis pathway is enriched for differentially expressed genes, with the DHCR7 gene exhibiting altered expression patterns [65]. Smith-Lemli-Opitz syndrome is caused by mutations in DHCR7, leading to a reduction of cholesterol synthesis and a buildup of 7-dehydrocholesterol [66].

There are several proteins that are highly associated with AMD. For example, all-trans retinaldehyde transporter (ABCA4), hemicentin (HMCN1), an extracellular protein involved in epithelial cell junction formation, multiple complement-related proteins (CFH and C3), age-related maculopathy susceptibility gene 2 (ARMS2) and apolipoprotein E (APOE) are all associated with the development of AMD [67]. APOE is a critical factor for the transport of lipids and cholesterol in the circulation, and the ApoE2 allele is a risk factor for AMD [68]. In addition, ApoE−/− mice exhibit pathophysiology similar to AMD, such as lipid deposits [69]. ApoE is expressed and released in cultured RPE cells, although we did not detect ApoE in our hTERT-RPE cell model.

A few sterol/steroid-related proteins that may play a role in AMD pathophysiology were identified in our dataset. For example, LRP1 is a receptor for APOE [67]. In this study, tBHP or γT exposure downregulated LRP1, suggesting that these stimuli may decrease the cellular uptake of cholesterol. Studies have determined that LRP1 expression was downregulated in an oxidative stress model of RPE damage in ARPE-19 cells and in a mouse model of AMD [67]. In addition, in human eyes with retinopathies, LRP1 expression was reduced in RPE-Bruch’s membrane-choriocapillaris complex and choroidal stroma [70].

TSPO is also downregulated by tocopherol treatments, and this study suggests that cholesterol/sterol import into mitochondria may be decreased. Others have suggested the role of cholesterol as a risk factor for AMD, which has generated an interest in focusing on cellular cholesterol efflux by increasing TSPO activity or by TSPO overexpression in RPE cells [35].

Our study also identified SCP2 as a differentially expressed protein in response to tBHP and tocopherols. SCP2 gene mutations are involved in the occurrence of Zellweger syndrome, which is characterized by cellular deficiency in peroxisomes with reduced synthesis of bile acids [71]. While there is no direct evidence that SCP2 plays a role in AMD, SCP2 knockout mice exhibit deficiencies in bile acid side chain cleavage and accumulation of phytanic acid. In addition, a patient with a mutation in SCP2 exhibited increased concentrations of pristanic and phytanic acid, leading to RPE dysfunction [71]. We also detected ApoL2, which is upregulated in response to oxidative stress. ApoL2 gene variants are associated with schizophrenia and substance abuse. To our knowledge, ApoL2 presence and expression have not been reported in RPE cells to date.

Other proteins identified in this study may play a role in Alzheimer’s disease (AD) and Parkinson’s disease. For example, LRP1 is critical for alpha 2-macroglobulin-mediated removal of secreted beta-amyloid, a major constituent of plaques in AD patients [72]. LRP1 gene expression diminishes with age and is also reduced in brain tissue from AD patients [72].

TSPO localizes to the outer mitochondrial membrane, where it reduces the opening of the mitochondrial permeability transition pore (mPTP) [73]. Opening of the mPTP can cause the onset of apoptosis by releasing the BCL2 family and cytochrome C proteins [73]. In AD, TSPO is upregulated to reduce neurodegeneration as AD progresses by elevating neuroprotective steroid synthesis, reducing β-amyloid toxicity and decreasing oxidative stress [74]. Furthermore, preclinical and early clinical studies indicate that ligands of TSPO exhibit anxiolytic and antidepressant effects by generating neurosteroid synthesis [74].

This study identified over twenty proteins involved in sterol transport and metabolism that were up- or downregulated following either oxidative stress, antioxidant exposure or both. Most proteins upregulated or downregulated by oxidative stress were similarly upregulated or downregulated by tocopherol exposure, suggesting that tocopherols may induce changes in RPE cells, which may allow them to better handle oxidative stress. The proteins identified in this study may represent potential therapeutic targets that protect RPE cells from oxidative damage and possibly AMD pathophysiology.

## 4. Materials and Methods

### 4.1. Cell Culture and Treatments

The human telomerase reverse transcriptase-overexpressing RPE (hTERT-RPE) cells (ATCC CRL-4000) were used for treatments and proteomics experiments. Cells were grown in DMEM:F12 with 10% fetal bovine serum and 10 μg/mL gentamicin and grown to confluence prior to treatments. The tocopherols, αT and γT (Millipore-Sigma, Burlington, MA, USA), in DMSO, were used in media at 100 μM. hTERT-RPE were treated with αT or γT (or DMSO vehicle) for 24 h before exposure to tert-butyl hydroperoxide (tBHP, 100 μM) or vehicle (water) control for 24 h as described previously [17].

### 4.2. Sample Preparation for Mass Spectrometry

The hTERT RPE cells from two experiments (biological replicates) were collected and lysed in RIPA buffer containing DNase as previously described [17]. Briefly, cell lysates were prepared for Trypsin digestion using a filter-aided sample preparation method. Resulting tryptic peptides from each condition from the two experiments were labeled individually with unique Tandem Mass Tags (TMT, Thermo Fisher Scientific, Waltham, MA, USA) and then recombined for multiplex TMT quantitation at approximately equimolar ratios, creating two mixed TMT samples, one mixture for each of the two experiments. The two TMT mixes were subjected to multidimensional chromatography (MudPIT) starting with offline fractionation using basic reversed phase separation in preparation for online LCMS of these fractions as described [17].

### 4.3. LCMS Data Acquisition and Database Searches/TMT Quantitation

MudPIT fractions were analyzed on a Fusion Lumos Orbitrap MS with SPS MS3 quantitation of TMT reporter ions and on a QExactive MS system with MS2 quantitation of TMT reporter ions. Details of the acquisition parameters and subsequent database search parameters are described in Duncan et al., 2023 [17]. In summary, the resulting data files were searched together using Proteome Discoverer 2.5 against Human Proteome UniProt UP000005640 (77,895 protein sequences, 28 June 2021) and a contaminants database—both were included as target databases. The reversed sequence human and contaminants databases were also searched (percolator node) for false discovery rate (FDR) calculations within Proteome Discoverer. The mass spectrometry proteomics data have been deposited to the ProteomeXchange Consortium via the PRIDE [75] partner repository with the dataset identifier PXD039513 and 10.6019/PXD039513.

### 4.4. Data Reconciliation and Organization

This manuscript contains new analyses carried out on the larger dataset generated from the same proteomics runs described for the first time in Duncan et al. 2023 [17]. As a result, we did not provide the same detailed sample preparation and MS/MS proteomics procedure as it was already described in adequate detail elsewhere [17].

In brief, Proteome Discoverer data were converted to Microsoft Excel 2016 for further analyses. Contaminant proteins and recognizable duplicate identifications were removed, and the average abundance values were determined for treatment group pairs (biological replicates), which were utilized for the assignment of absent values. The standard deviation was determined and utilized to identify proteins that were altered the least among groups.

Scientific literature and other databases (PubMed GeneCards^®^, Entrez and UniProt) were searched to identify proteins recognized to be expressed in RPE cells and their suitability as housekeeping proteins for data normalization, as described in more detail elsewhere [17]. The average protein abundance (normalized), variance and standard deviation were determined for every protein in the experimental groups. A Student’s *t*-test (two-tailed with unequal variance) was carried out for treatment group ratio comparisons. The fold-change values were determined in EnrichR and (PubMed) Kyoto Encyclopedia of Genes and Genomes (KEGG) pathway analyses.

### 4.5. Gene Ontology (GO) and Functional Category Analyses

The EnrichR program was used with gene names and fold-change values to conduct a Genome-Wide Enrichment analysis utilizing the common databases “KEGG_2021_Human”, “GO molecular function”, “GO biological process”, “GO cellular component”. Pathway analysis and KEGG pathway mapping were conducted using an R script described in more detail elsewhere [17]. Software-based analysis, using R, EnrichR and KEGG (all recent versions as of 2022; [17]), was implemented for GO and pathway classification. In addition, proteins based on broad biological functions, such as sterol uptake, sterol/steroid hormone transport and sterol/steroid hormone metabolism, were assembled manually from curated protein databases (GeneCards, Entrez and UniProt) and literature.

We identified 22 differentially expressed proteins involved in sterol/steroid hormone function following the induction of oxidative stress. The differentially expressed proteins were divided into three groups based on functional class: sterol uptake, sterol transport and sterol metabolism. The pertinence of identified proteins to cholesterol/sterol uptake, transport or metabolism was verified by searching gene/protein databases, including GeneCards^®^, Entrez and UniProt.

### 4.6. Statistical Analysis

A technical replicate, in addition to a biological replicate, was carried out in this study. Each biological replicate lysate was used (separately) for TMT MudPIT, with each being repeated twice as a common technical replicate.

For each treatment group, the protein abundance was normalized by dividing by the abundance of the control. Individual protein expression level differences between groups were calculated using a Student’s *t*-test in Microsoft Excel^®^. *p*-values of ≤0.05 (*) and ≤0.01 (**) were considered significant and highly significant, respectively. The *t*-tests were specifically performed by comparing the normalized replicate averages for a particular protein in the control group (veh-NT) versus the normalized replicate average for the same protein in the experimental group (e.g., veh-tBHP). A significance cutoff of 0.05, which is 0.025 per tail, was used. 

## Figures and Tables

**Table 1:** The effect of tBHP exposure on cellular cholesterol uptake.

	Fold-Change	*p*-Value
LRPAP1	20.4	0.001
APOL2	17.0	0.022
LRP10	−6.4	0.001
LRP1	−15.1	0.003

**Table 2:** The effect of tocopherol exposure on cellular cholesterol uptake.

	α-Tocopherol		γ-Tocopherol	
	Fold-Change	*p*-Value	Fold-Change	*p*-Value
LRPAP1	19.9	0.0001	17.5	0.002
*LRP10*	*−0.1*	*0.157*	−4.8	0.001
*LRP1*	*−3.7*	*0.092*	−9.2	0.001

**Table 3:** The effect of tBHP exposure on intracellular sterol transport.

	Fold-Change	*p*-Value
SCP2	42.8	0.002
CAV1	20.6	0.003
OSBP	6.6	0.020
OSBPL1A	−5.1	0.011
OSBPL9	−11.6	0.014

**Table 4:** **The effect of tocopherol exposure on** intracellular sterol transport.

	α-Tocopherol		γ-Tocopherol	
	Fold-Change	*p*-Value	Fold-Change	*p*-Value
SCP2	31.5	0.012	50.5	0.001
OSBP	5.4	0.049	13.0	0.006
OSBPL3	−15.9	0.032	−14.9	0.037
TSPO	−41.0	0.0075	−43.0	0.006

**Table 5:** The effect of tBHP exposure on sterol metabolism.

	Fold-Change	*p*-Value
DHRS7	22.03	0.006
AKR1B1	19.5	0.00004
HSD17B10	−8.6	0.010
LSS	−9.5	0.013
HSD17B11	−13.6	0.048
LBR	−17.2	0.017
DHCR7	−20.5	0.010
CYP1B1	−21.5	0.002
CYP51A1	−27.7	0.011

**Table 6:** The effect of tocopherol exposure on sterol metabolism.

	α-Tocopherol		γ-Tocopherol	
	Fold-Change	*p*-Value	Fold-Change	*p*-Value
AKR1B1	25.6	0.000002	18.9	0.00004
*LSS*	*−3.1*	*0.367*	−16.3	0.001
HSD17B10	−6.0	0.033	−7.7	0.016
*HSD17B11*	*−7.1*	*0.234*	−14.0	0.045
*EBP*	*−9.5*	*0.116*	−15.8	0.026
DHRS4	−12.2	0.044	−16.0	0.017
LBR	−21.6	0.005	−28.4	0.001
DHCR7	−22.3	0.011	−25.7	0.003

**Table 7:** Effect of sequential tocopherol-tBHP exposures on steroid hormone uptake, transport and metabolism.

	αT-tBHP/veh-tBHP		γT-tBHP/veh-tBHP	
	Fold-Change	*p*-Value	Fold-Change	*p*-Value
LRPAP1	−8.3	≤0.01	−3.7	0.028
*LRP1*	*−2.8*	*0.055*	*−2.7*	*0.056*
SCP2	−3.9	0.022	−5.6	0.012
CAV1	−20.8	≤0.01	−7.8	0.029
OSBPL1A	*2.3*	*0.151*	5.3	0.038
TSPO	−36.6	≤0.01	*−2.2*	*0.210*
DHRS7	−6.5	0.027	*0.5*	*0.200*
*AKR1B1*	*−3.6*	*0.380*	*4.0*	*0.053*
EBP	*−1.5*	*0.693*	3.6	≤0.01

## Data Availability

The article encompasses the original contributions from the study. For additional queries, please reach out to the respective author.
